# Cryptosporidiosis outbreak in Amazonia, French Guiana, 2018

**DOI:** 10.1371/journal.pntd.0010068

**Published:** 2022-01-31

**Authors:** Estelle Menu, Emilie Mosnier, Arnaud Cotrel, Loic Favennec, Romy Razakandrainibe, Stéphane Valot, Denis Blanchet, Frédéric Dalle, Damien Costa, Mélanie Gaillet, Magalie Demar, Franck de Laval

**Affiliations:** 1 Laboratoire Hospitalo-Universitaire de Parasitologie-Mycologie, Centre Hospitalier Andrée-Rosemon, Cayenne, French Guiana; 2 Laboratoire Hospitalo-Universitaire de Parasitologie-Mycologie, Institut Hospitalo-Universitaire, Méditerranée Infection, Marseille, France; 3 Aix Marseille Université, IRD, AP-HM, IHU-Méditerranée Infection, UMR Vecteurs–Infections Tropicales et Méditerranéennes (VITROME), Marseille, France; 4 Unité des Maladies Infectieuses et Tropicales (UMIT), Centre Hospitalier Andrée Rosemon, Cayenne, French Guiana; 5 Sciences Economiques & Sociales de la Santé & Traitement de l’Information Médicale, Aix Marseille University, INSERM, IRD, SESSTIM, Marseille, France; 6 French Armed Forces Health Service in French Guiana, Cayenne, French Guiana; 7 CNR-LE Cryptosporidioses, Laboratoire de Parasitologie Mycologie, CHU Rouen, Rouen, France; 8 University of Medicine Pharmacy Rouen EA ESCAPE 7510, Rouen, France; 9 Laboratoire de Parasitologie Mycologie, Laboratoire Collaborateur du CNR-LE Cryptosporidioses, CHU Dijon, Dijon, France; 10 Ecosystèmes amazoniens et Pathologie Tropicale, Université de la Guyane, Cayenne, French Guiana; 11 UMR PAM, Equipe VAlMiS, Université Bourgogne Franche-Comté, Dijon, France; 12 Pôle des Centres Délocalisés de Prévention et de Soins, Centre hospitalier Andrée Rosemon, Cayenne, French Guiana; 13 SSA, Service de Santé des Armées, CESPA, Centre d’épidémiologie et de santé publique des armées, Marseille, France; Istituto Superiore di Sanità, ITALY

## Abstract

**Background:**

Cryptosporidiosis outbreaks in South America are poorly documented. In March 2018, 51 cases of cryptosporidiosis were reported in Maripasoula, a village located in a remote forest area along the border between Surinam and French Guiana.

**Method:**

To identify the origin of the epidemic, we performed epidemiological, microbiological, and environmental investigations. Only the cases involving diarrhoea and *Cryptosporidium*-positive stool were considered as *bona fide*, while cases involving diarrhoea and close contact with a confirmed case were classified as “possible”.

**Results:**

We identified 16 confirmed cases and 35 possible ones. Confirmed cases comprised nine children (median age of 18 months, range: 6–21), one immunocompromised adult and six soldiers. One child required a hospitalisation for rehydration. All 16 *Cryptosporidium* stools were PCR positive, and sequencing of the *gp60* gene confirmed only one *Cryptosporidium hominis* subtype IbA10G2. Tap water consumption was the only common risk factor identified. Contamination of the water network with *Cryptosporidium parvum* subtype IIdA19G2 was found.

**Conclusion:**

Water quality is a major public health issue in Amazonian French Guiana, especially for population at risk (children, people with comorbidity, travelers). For them, alternative water supply or treatment should be implemented.

## Introduction

The apicomplexan protozoan *Cryptosporidium* is distributed worldwide and is one of the most common waterborne pathogens [1]. It is the second leading cause of diarrheal death for children under the age of five, causing 12% of the total diarrhoea mortality burden [2]. Its transmission is fecal-oral and can occur through the ingestion of oocysts from untreated water (drinking or recreational) or food and by contact with infected persons or animals [3], with an incubation period of 5 to 7 days [4]. Ingestion of faecally-contaminated water has been responsible for large outbreaks [4,5]. Symptoms are non-specific and include diarrhoea, nausea, vomiting, lack of appetite and cramps, which complicates diagnosis of immunocompetent patients [6]. Symptoms may be protracted and severe in immunocompromised patients and in children–for whom they are frequently associated with severe watery diarrhoea and dehydration [7].

*Cryptosporidium hominis* and *C*. *parvum* are the primary causative agents of human cryptosporidiosis, but their prevalence varies in different regions of the world [3]. Transmission had already been reported along the Maroni river, a river flowing in the Amazonian forest between French Guiana and Surinam, although the mode of contamination was not well understood [8]. Maripasoula is an insulated border town between French Guiana and Surinam, an hour by plane or three days by canoe from the nearest road. A significant number of Maripasoula’s inhabitants live in precarious conditions. Epidemiological surveillance in Maripasoula consists in the monitoring of inhabitants requiring care for diarrhoea in the Delocalised Centre for Prevention and Care (CDPS). In March 2018, the physician in charge for infectious diseases at the CDPS alerted the Regional Health Authority in Cayenne about three successive immunocompetent children having cryptosporidiosis.

On April 24, 2018, epidemiological surveillance by the French army detected an increase in the incidence of cryptosporidiosis. This outbreak allowed further understanding of cryptosporidiosis transmission along the Maroni river, by identifying the mode of contamination and further developing appropriate countermeasures.

## Materials and methods

### Ethics statement

The study was approved by the ethics committee of the Andrée Rosemon Hospital, Cayenne (French Guiana). Patients’ medical charts were reviewed to collect demographic, exposure, clinical, biological, microbiological, and outcome data *via* anonymised and standardised forms. The collected database of medical charts was declared to the French Data Protection Authority (CNIL), no.1939018.

### Case definition

**Fig 1:**
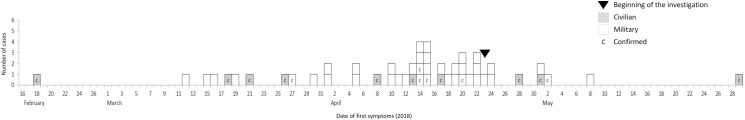
Epidemiological curve showing the distribution of confirmed (C) and possible cases over time (January 1—May 31, 2018).

A “confirmed case” was defined as a patient living or stationed in Maripasoula between January 1 and May 31, 2018, with gastrointestinal illness (diarrhoea or nausea or vomiting or abdominal pain), and a *Cryptosporidium*-positive stool test. Other patients with diarrhoea and in close contact with a confirmed case (same household and time) were defined as “possible cases” without further stool examination (Fig 1). Finally, “control cases” were subjects with similar environmental exposure (Maroni banks) but who did not present any symptom.

### Population study

**Fig 2:**
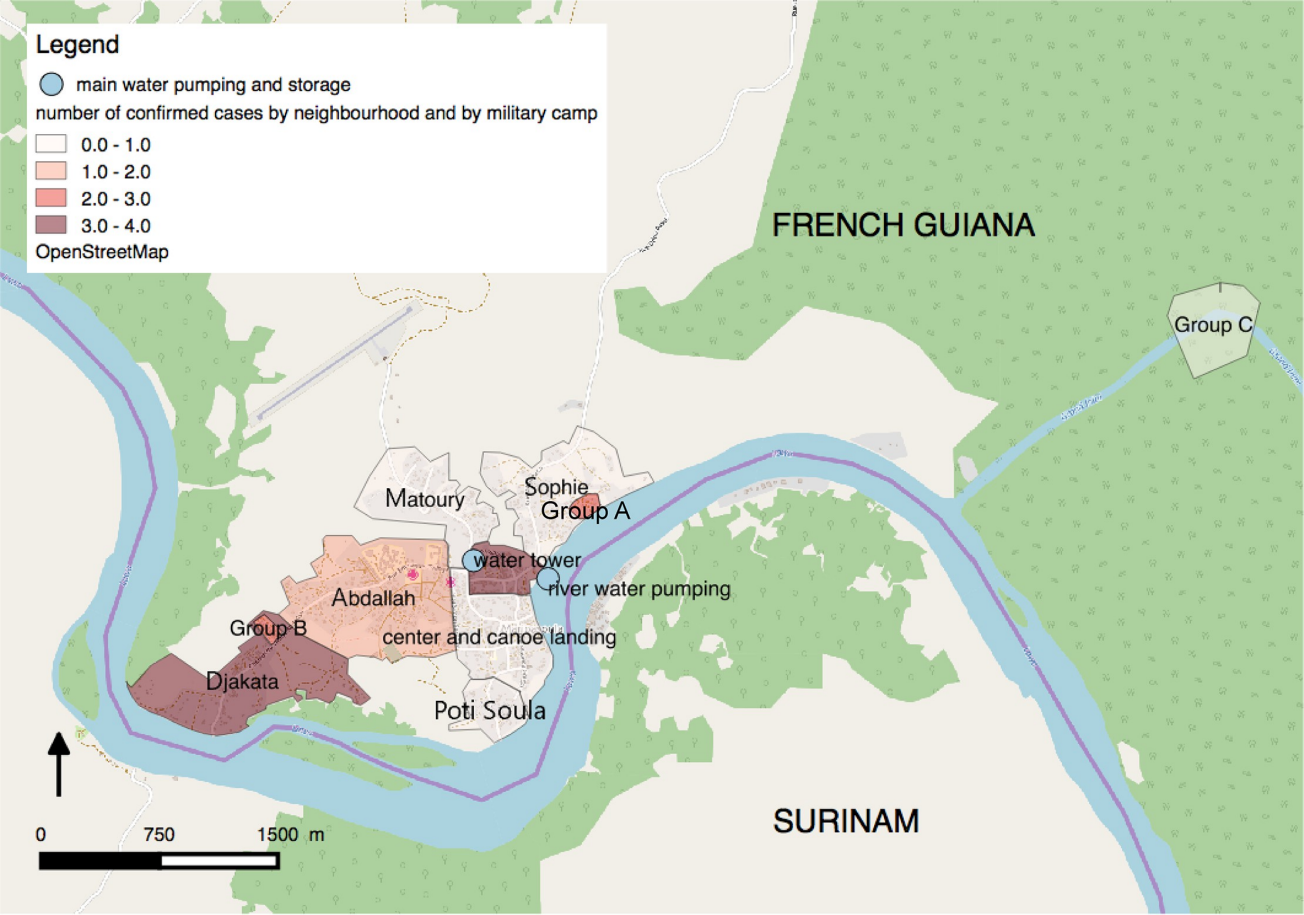
Map of Maripasoula. Spatial distribution of cases and the water catchment area. The map was generated in QGIS software (QGIS Development Team (2020). QGIS Geographic information System. Open Source Geospatial Foundation Project. http://qgis.osgeo.org). The data on the map comes from the CDPS database and the military health service.

To determine the most likely common source of contamination, we collected information from confirmed cases’ medical records regarding demographic characteristics, clinical manifestations, and laboratory results (Table 1). In addition, a questionnaire was used to collect information regarding food exposures, water consumption, contact with animals and relationship/contact between patients. Places of residence were also collected and placed on a map to identify areas of interest (Fig 2). Military and police personnel were fully available, and a retrospective cohort study was possible among confirmed and control cases. Poisson regression enabled us to identify the most probable source of contamination.

**Table 1:** Confirmed cases: Demographic characteristics, clinical manifestations and laboratory results. MPA: Maripasoula.

					*Civilians*								*Soldiers*			
	*Case 1*	*Case 2*	*Case 3*	*Case 4*	*Case 5*	*Case 6*	*Case 7*	*Case 8*	*Case 9*	*Case 10*	*Case 1*	*Case 2*	*Case 3*	*Case 4*	*Case 5*	*Case 6*
***Date of first symptoms (2018)***	18 February	18 March	21 March	26 March	8 April	13 April	17 April	28 April	1 May	29 May	27 March	14 April	14 April	15 April	20 April	02 May
***length of illness***	1 day	13 days	26 days	2 days	11 days	22 days	3 days	Unknown	8 days	68 days	17 days	Unknown	19 days	3 days	5 days	4 days
***Age***	12 months	6 months	21 months	18 months	19 months	19 months	11 months	12 months	60 years	20 months	34 years	48 years	22 years	30 years	23 years	21 years
***Sex***	Male	Female	Female	Female	Male	Male	Male	Male	Male	Male	Male	Male	Male	Male	Male	Male
***Urban area***	MPA	MPA	MPA	MPA	MPA	MPA	MPA	MPA	MPA	MPA	MPA	MPA	MPA	MPA	MPA	MPA
***District***	Abdallah	Montagne	Montagne	Abdallah	Djakata	Djakata	Montagne	Abdallah	Abdallah	Sophie	Abdallah	Abdallah	Sophie	Abdallah	Sophie	Sophie
***Comorbidity***	No	No	No	No	No	No	No	Yes	Yes	No	No	No	No	No	No	No
***Immunosuppressive factors***	No	No	No	No	No	No	No	Yes ^(1)^	Yes ^(2)^	No	No	No	No	No	No	No
***Symptoms***																
***Fever*** ^***(3)***^	Yes	Yes	Yes	No	Yes	No	No	No	No	No	Yes	No	No	No	No	No
***Diarrhoea***	Yes	Yes	Yes	Yes	Yes	Yes	Yes	Yes	Yes	Yes	Yes	Yes	Yes	Yes	Yes	Yes
***Vomiting***	Yes	No	No	No	Yes	No	No	No	Yes	Yes	No	No	Yes	Yes	No	No
***Hospitalization***	No	No	No	No	No	No	No	Yes	No	Yes	No	No	No	No	No	No
***Nitazoxanide TUA***	No	No	No	No	No	No	No	Yes	No	No	No	No	No	No	No	No
***Coinfection***	No	Yes	Yes	No	No	No	No	No	No	No	No	No	No	No	No	No
***Sample date***	-	21 March	22 March	-	-	-	-	-	-	-	-	-	-	-	-	-
***Species***	-	*Salmonella* spp.	Norovirus	-	-	-	-	-	-	-	-	-	-	-	-	-
***Consumption of regular tap water***	Yes	Yes	Yes	Yes	Yes	Yes	Yes	Yes	Yes	Yes	Yes	Yes	Yes	Yes	Yes	Yes
***Contact with animals***	No	No	No	No	No	No	No	No	No	No	No	No	No	No	No	No

MPA: Maripasoula

^(1)^ Malnutrition

^(2)^ Kidney transplant recipient

^(3)^ Temperature > 38°C

**Fig 3:**
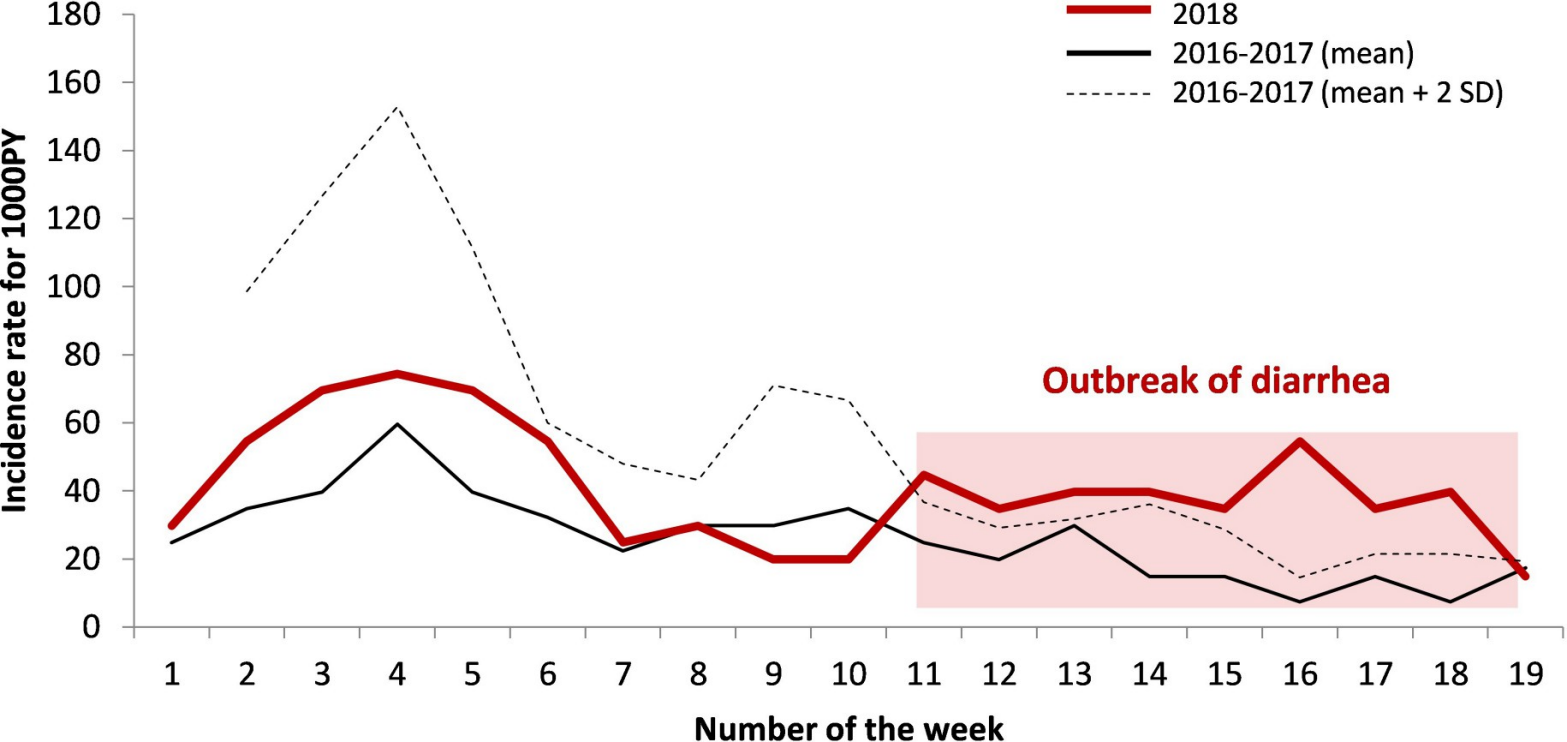
Incidence of diarrhoeal cases in the Maripasoula health centre in 2018 compared to the two previous years (for 1,000 persons-year). The number of weeks correspond to the year 2018 i.e. from January 1 to May 13, 2018.

Data on diarrhoeal cases from the Maripasoula CDPS monitoring system were analysed during the outbreak and compared with the two previous years (Fig 3). We used the data from the French national institute for statistics and economic studies (Institut national de la statistique et des études économiques–INSEE) [9] concerning the official population of the municipality of Maripasoula which was accurate as of January 1, 2016 (12,798) to calculate the attack rate.

### Fecal sample collections and parasite detection

**Fig 4:**
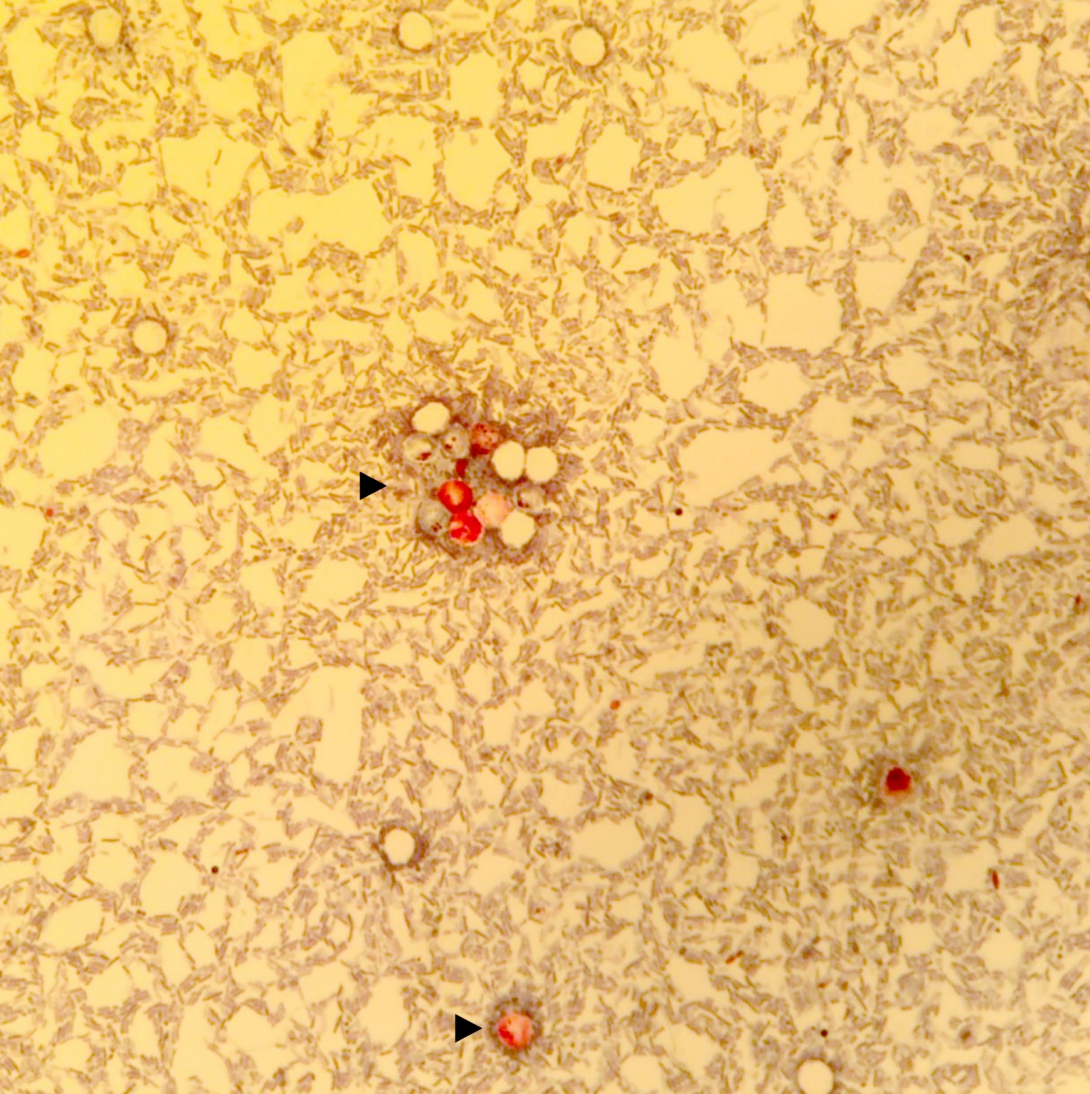
Stool sample after modified Ziehl-Neelsen stain. *Cryptosporidium* spp. oocysts are stained fuchsia pink (500x magnification). Isolated or grouped oocysts are indicated by black arrows.

Between, January 1 and May 31, 2018, 103 faecal samples from Maripasoula were examined at the Parasitology Laboratory of Andrée Rosemon Hospital, Cayenne. A Bailanger’s concentration method and Kato-Katz technique were carried out for intestinal parasites identification. For the identification of *Cryptosporidium* oocysts, a modified Ziehl–Neelsen stain (Fig 4) was performed on the concentration pellet of the enrichment performed with the Paraprep L SAF kit (Diamodal, Vienna, Austria) [10,11]. To account for the possibility of other infectious aetiologies, stool specimens were also tested using bacterial culture and rapid diagnosis test screening for adenovirus, rotavirus (Adeno-Rota color, Servibio, Les Ulis, France) and norovirus (Norovirus GenI and GenII, Servibio, Les Ulis, France).

All stool samples were sent to the French national reference center in France. DNA extraction was directly performed from received stools without any previous concentration. Two molecular methods were performed after DNA extraction according to manufacturer’s instructions using a NucliSENS easyMAG device (bioMérieux, Marcy l’Etoile, France) [12]. An in-house real-time polymerase chain reaction (RT-PCR) assay was set up to enable the detection and identification of the most common *Cryptosporidium* species/genotypes based on differences in the melting temperatures of the PCR-probe complexes [13]. The technique was validated using DNA from characterised *Cryptosporidium* genus and positive controls for *C*. *parvum* and *C*. *hominis* [14]. All isolates were further subtyped using a *gp60*-based tool. GP60 subtypes were identified according to the protocol described by Sulaiman et al. [15]. Briefly, a nested PCR was performed using primers: AL3531 (5’-ATAGTCTCCGCTGTATTC-3’)/AL3533 (5’-GAGATATATCTTGGTGCG-3’) and secondly AL3532 (5’-TCCGCTGTATTCTCAGCC-3’)/LX0029 (5’-CGAACCACATTACAAATGAAGT-3’). Thermocycling conditions were: 94°C for 3 min, followed by 40 cycles of 94°C for 45 s, 54°C for 45s and 72°C for 60 s and a final step at 72°C for 7 min. Sequencing was performed using an AB3500 automated sequencer (Applied Biosystems, Illkirch, France) [16]. The nucleotide sequences obtained categorised *C*. *parvum* and *C*. *hominis* to many families of subtypes, by aligning the *gp60* sequences obtained and reference sequences retrieved from GenBank.

### Water collection and parasite detection

The drinking water in Maripasoula was pumped from two main sources: the Maroni river (≈70%) and the ground (≈30%). These sources were then mixed at the water tower and distributed throughout the town. Before storage and distribution, the water from the Maroni was sand filtered, flocculated, and chlorinated (>1.0 mg/L). The water from the ground was not treated and mixed with Maroni treated water. The wells were temporarily shut down between December 2017 and March 2018, but ground water was nevertheless sampled. Two samples were taken from the water tower on May 29, 2018: untreated water coming from the Maroni river (100 L) and post-treatment water (100 L). A peripheral tap water sample was taken from a water fountain (100 L) in the police settlement. For each sample, water was filtered through a standardised 1-micron filter Envirocheck sampling capsule (PALL, Saint-Germain-en-Laye, France). All samples were sent to the national reference center in France. Each received Envirocheck sampling capsule was eluted twice using 120 mL of an elution solution composed of 750 μl of Tween 80 (previously 10-fold diluted in PBS) (Sigma-aldrich, Vienne, Austria) and 750 μl of antifoam B emulsion (previously 10-fold diluted in PBS) (Sigma-aldrich, Vienne, Austria) in PBS 1X (Sigma-aldrich, Vienne, Austria) qsp 500mL.

Eluates were centrifugated at 2500 rpm for 30 minutes at +4°C. The oocysts potentially encountered in pellets of centrifugation were isolated with immunocapture using magnetic beads covered with specific antibodies from the walls of *Cryptosporidium* spp. oocysts (Isolate *Cryptosporidium*, TCS Bioscience Ltd, Buckingham, United Kingdom). They were then eluted (100 μl final) following the manufacturer’s recommendations. Ten microliters of the suspension were used for microscopic observation of the oocysts with immunofluorescence methods using a mixture of anti-*Cryptosporidium* monoclonal antibodies labeled with fluorescein isothiocyanate (FITC) (Crypto-Cell IF; Cellabs, Australia), following manufacturer’s recommendations.

DNA extraction from environmental samples was conducted on the 90μL remaining suspensions using QIAmpPowerFecal DNA Kit (Qiagen, Hilden, Germany) following the manufacturer’s recommendations. An RT-PCR amplifying 166 bp from the LIB13 locus was carried out to detect and differentiate the species present according to method described by Hadfield et al. [17]. Sequencing was performed as previously described for stool investigations [16].

## Results

### Outbreak and patients’ description

Between January 1 and May 31, 2018, we diagnosed 16 confirmed cases: nine in immunocompetent children, six among military and police personnel, and one was a 60-years-old immunocompromised man (a kidney transplant patient) (Fig 1 and Table 1). In addition, there were 35 possible cases among military and police personnel.

The global attack rate was 0.12% (16/12,798). It was 0.94% (9/962) in 0-5-aged-children, and 26% (41/157) among military and police personnel.

The median age was 18 months for children [range 6–21 months], and 28 years for military and police personnel [range 20–50 years]. One child had comorbidity and was hospitalized for severe malnutrition, and two needed hospitalization to be rehydrated, one of whom was treated with Nitazoxanide. No adult patient requires hospital care. Symptoms were diarrhoea (9/9), fever (4/9) and vomiting (3/9) among children, and diarrhoea (6/7), vomiting (5/7), abdominal pain (4/7), headache (2/7) and fever (1/7) among adults; median duration of symptoms were 11 and five days respectively. All patients eventually recovered.

*Cryptosporidium hominis* subtype IbA10G2 was found in all stool samples (Genbank accession number of the representative sequence OK032157). Two children had co-infections with *Salmonella* spp. and norovirus, respectively.

Confirmed civilian cases came from different districts of the city and did not share obvious similarities besides tap water consumption (Fig 2).

### Retrospective cohort study

Military and police personnel were divided into three groups. Group A and B stayed in two different settlements in Maripasoula, without any contact, and with different food supply except the consumption of tap water. Group C was 30 minutes upstream from Maripasoula, their food was the same as Group B, but water was supplied from bottled-water. The retrospective cohort study showed that tap-water consumption was at risk for cryptosporidiosis (RR = 9.9, 95% CI = [2.5–65.7], Table 2). None of them had any contact with animals or with recreational water in the Maroni during the incubation period.

**Table 2:** Poisson regression about cryptosporidiosis factors of exposure, results from the retrospective cohort study among military and police personnel in Maripasoula (n = 157), French Guiana, 2018.

Factor of exposure	Cases	Total number exposed	Univariate			Multivariate		
			RR	95%CI	p	RR	95%CI	p
Tap water (DA = 157)								
no	**5**	**72**	**ref**					
yes	**41**	**85**	**1.8**	**1.5–2.3**	**<10** ^**−5**^	**9.9**	**2.5–65.7**	**0.004**
Bottled water (DA = 132)								
no	12	28	ref					
yes	24	104	0.7	0.5–1.0	0.04	0.6	0.3–1.2	0.12
Local food (DA = 155)								
no	29	129	ref					
yes	15	26	1.8	1.2–2.9	<10^−3^	1.3	0.6–3.0	0.43
Raw vegetables, fruits (DA = 153)								
no	15	95	ref					
yes	27	58	1.6	1.2–2.0	<10^−3^	1.2	0.6–3.0	0.62
Contact with animals (DA = 157)								
no yes	0 0	0 0	-	-	-	-	-	-
Recreational water (DA = 157)								
no yes	0 0	0 0	-	-	-	-	-	-

DA = data available; ref: reference

### Investigation of the drinking water system

According to our investigation, Maroni’s surface water was at considerable risk of pollution: water was pumped from the middle of downtown Maripasoula, the water catchment point was located at few meters away from the riverbanks, and the security perimeter around the water catchment point was not respected, highly exposed to contamination from dirty and runoff waters of the town. A review of the water treatment system (sand filtration and chlorination) determined that it was ineffective in case of substantial contamination.

No oocyst was detected by microscopy. Two samples of the three were found positive by molecular methods for *C*. *parvum*: the post-treatment water (Ct: 38.03) and the drinking water (Ct: 39.01) from Maripasoula’s water distribution system. Ground water was found to be negative. Only one sample could be analysed (the post-treatment water, subtyping using *gp60* gene), and revealed the presence of *Cryptosporidium parvum* subtype IIdA19G2 (Genbank accession number of the representative sequence OK032156).

### Outbreak controls measures

In June 2018, the company responsible for the water supply in Maripasoula improved the water treatment system, specifically regarding the maintenance and repair of pumps, pre-treatment pH-adjustment to optimise the coagulation process and further improve water clarification, sand filters and tank cleaning (resulting in a temporary increase in water chlorination). Displacement of the water catchment point was planned to move further upstream on the Maroni river. Military camps were supplied with bottled water from May 24 to the end of June 2018, then UV lights system were installed. Only a new case of cryptosporidiosis was reported in September 2018. Stool sample genotyping revealed *C*. *hominis* subtype IbA10G2, the same genotype found during the epidemic. The sample was taken from a 9-month-old male who had presented diarrhoea for 24 hours. His only risk factor was the consumption of soups prepared with tap water, although the hypothesis of contact with an asymptomatic carrier could not be ruled out as a source of exposure.

## Discussion

This outbreak of cryptosporidiosis in French Guiana was an opportunity to better understand cryptosporidiosis transmission in the Amazonian forest ecosystem. In the present study, the identification of *C*. *hominis* subtype IbA10G2 in all the stool samples suggested a common source of contamination. This subtype is the most commonly identified subtype in waterborne cryptosporidiosis outbreaks worldwide [18–20], and it has be shown to be more virulent than other subtypes resulting in intensive transmission [20]. Data in South America remains scarce. Environmental studies carried out in Colombia and Brazil have reported the presence of a great spectrum of *Cryptosporidium* spp. in raw and treated water as well as in animal reservoirs, including *C*. *parvum* [21–23]. Other molecular studies have reported genetic diversity of *Cryptosporidium* in human stool samples from Argentina, Brazil and Peru, each time *C*. *hominis* subtype IbA10G2 was identified; including two outbreaks in Peru and Columbia [20,24–27]. Therefore, it is the predominant subtype in South America. In our study, available evidence indicated a waterborne source of contamination through the public water supply network. Tap water was the unique common link between civilians, military personnel, and police personnel. Results of the retrospective cohort study were concordant with this statement. Human transmission was not suspected as none of the groups had contacts together. Contact with animals was not reported by any patients; which was in keeping with the subtype involved which excluded zoonotic transmission [28]. These findings were consistent with the deficiencies found by the investigation into the drinking water system. There must have been an initial contamination of the resource (which was exposed to the effluents), followed by a failure in the treatment system. Indeed, the environmental investigation found contamination of the water network with *C*. *parvum* subtype IIdA19G2. Isolation of a different species from that found in the stool of the confirmed cases can be explain by the sampling delay, two months after the outbreak, knowing that the concentration of cryptosporidia is highly variable in the environment. Isolating these protozoa from the water was laborious, there was no standardised method for catching and detecting parasites at the environmental level [11,29]. Molecular and genetic analyses have now been developed [30], but these techniques are dependent on the quality and number of the samples received. Unfortunately, only four samples could be processed because of logistical issues: we used all the filters available, and we had to send samples from the depths of the Amazonian forest to metropolitan France within 72 hours at 4°C, which is costly. Moreover, the detection threshold of the PCR we used was 100 times better for *C*. *parvum* than for *C*. *hominis* [31] which can explain why only *C*. *parvum* was found in the water samples. Due to all these constraints, the contamination of the water network as the source of the outbreak remained the main hypothesis.

This outbreak highlighted the need to enhance drinking water treatment. Slifko et al. reported that *Cryptosporidium* was resistant to disinfection with chlorine at concentrations typically applied in drinking water treatment plants (2 to 6 mg/L) [32]. However, alternative methods of disinfection are available such as pulsed UV light [33], or slow sand filtration [34], which is a pragmatic but effective method. The security of the resource is also primordial and should be moved upstream Maripasoula. However, in order to prevent these infections in low-resource populations, one of the inexpensive public advices for residents and visitors to these areas is to boil the water for one to three minutes to eliminate *Cryptosporidium* oocysts [35].

In this outbreak, the children affected by the epidemic were under two years old [36,37]. *Cryptosporidium* is among the leading causes of moderate-to-severe diarrhoea in children under two years-old, and may be under recorded when asymptomatic or associated with other pathogens [38]. Severe cases generally involve immunocompromised patients, infants or patients with comorbidities [38,39]. On the other hand, the military and police personnel were "naive" adults arriving in a new environment, and it could have been their first contact with the pathogen [40]. They were therefore more vulnerable, explaining the high attack rate observed among them.

## Conclusion

We report the second epidemic of cryptosporidiosis reported in French Guiana since 2015 [8], with some severe cases among children, and a high attack rate among temporary residents (here the military and police personnel). Water quality is a real public health problem in Amazonian forest, especially for population at risk (children, people with comorbidity, travelers). For them, alternative water supply or improved treatment should be implemented.

**Key learning points**

- This outbreak in the Amazonian forest highlights a waterborne transmission of *Cryptosporidium hominis* which is consistent with previously published data.
- Water quality and water treatment is a real public health issue in Amazonian forest, especially for populations at risk and “naïve” adults.
- For these particular populations it is necessary to set up means of prevention for cryptosporidiosis, such as bottled or boiled water consumption.

**Top five papers**

- Baldursson S, Karanis P. Waterborne transmission of protozoan parasites: review of worldwide outbreaks—an update 2004–2010. Water Res. 2011;45: 6603–6614.
- Checkley W, White AC, Jaganath D, Arrowood MJ, Chalmers RM, Chen X-M, et al. A review of the global burden, novel diagnostics, therapeutics, and vaccine targets for *Cryptosporidium*. Lancet Infect Dis. 2015;15: 85–94.
- Chappell CL, Okhuysen PC, Sterling CR, Wang C, Jakubowski W, Dupont HL. Infectivity of *Cryptosporidium parvum* in healthy adults with pre-existing anti-*C*. *parvum* serum immunoglobulin G. Am J Trop Med Hyg. 1999;60: 157–164.
- Li N, Xiao L, Cama VA, Ortega Y, Gilman RH, Guo M, et al. Genetic Recombination and *Cryptosporidium hominis* Virulent Subtype IbA10G2. Emerg Infect Dis. 2013;19: 1573–1582.
- Chyzheuskaya A, Cormican M, Srivinas R, O’Donovan D, Prendergast M, O’Donoghue C, et al. Economic Assessment of Waterborne Outbreak of Cryptosporidiosis. Emerg Infect Dis. 2017;23: 1650–1656.
